# Good performance in the management of acute heart failure in cardiogeriatric departments: the ICREX-94 experience

**DOI:** 10.1186/s12877-021-02210-0

**Published:** 2021-05-01

**Authors:** Emmanuelle Berthelot, Amaury Broussier, Thibaud Damy, Cristiano Donadio, Stephane Cosson, Xavier Rovani, Emmanuel Salengro, Gilles Billebeau, Richard Megbemado, Noomen Rekik, Christian Godreuil, Kevin Richard, Jason Shourick, Patrick Assayag, Joel Belmin, Jean Philippe David, Luc Hittinger, for the FINC-94 network

**Affiliations:** 1 Université Paris Sud, Le Kremlin-Bicêtre, France; 2 APHP, Department of Cardiology, Hopital Bicêtre, 78, rue du général Leclerc, 94270 Le Kremlin Bicêtre, France; 3 Université Paris Est, Créteil, INSERM, IMRB, Equipe CEpiA, F-94010 Créteil, France; 4 Department of Geriatrics, AP-HP, Henri-Mondor/Emile-Roux hospitals, F-94456 Limeil-Brevannes, France; 5 Department of Cardiology, heart failure and amyloidosis unit, Referral Center For Cardiac Amyloidosis, AP-HP, Henri-Mondor/Albert-Chenevier hospitals, F-94010 Créteil, France; 6 Department of geriatrics, AP-HP, Hôpital Charles Foix and Sorbonne Université, F-94200 Ivry-sur-Seine, France; 7 Hôpital privé Paul Dégine, 4 avenue Marx Dormoy, F-94500 Champigny-sur-Marne, France; 8 Centre Hospitalier de Villeneuve St Georges, 40 allée de la Source, F-94190 Villeneuve-Saint-Georges, France; 9 Hôpital Sainte Camille, 2 rue des Pères Camilliens, F-94360 Bry-sur-Marne, France; 10 Hôpital d’Instruction des Armées Bégin, 69 avenue de Paris, F-94160 Saint-Mandé, France; 11 AP-HP Centre hospitalier Chenevier, 40 rue de Mesly, F-94000 Créteil, France; 12 AP-HP, BioStatisticien, Paris Sud, Paris, France; 13 Department of Internal Medicine and Geriatrics, AP-HP, Henri Mondor hospitals, F-94010 Créteil, France

**Keywords:** Heart failure, Cardiogeriatrics, Prognosis, Profile, Risk factors

## Abstract

**Context:**

A growing number of elderly patients hospitalized for Acute Heart Failure (AHF) are being managed in cardiogeriatrics departments, but their characteristics and prognosis are poorly known. This study aimed to investigate the profile and outcome (rehospitalization at 90 days) of patients hospitalized for AHF in cardiogeriatrics departments in the Val-de-Marne area in the suburbs of Paris, and to compare them to AHF patients hospitalized in cardiology departments in the same area.

**Methods:**

Observational study, ICREX-94, conducted in seven cardiology departments in France and three specific cardiogeriatrics departments in Val-de-Marne.

**Results:**

A total of 308 patients were hospitalized for AHF between October 2017 and January 2019. During the 90 days following discharge, 29.6% patients were readmitted to the hospital. Compared with patients hospitalized in cardiology departments, patients in cardiogeriatrics departments were older (*p* < 0.001), less independent (living more often alone or in an institution) (*p* < 0.001), more often depressed (p < 0.001), had more often major neurocognitive disorder (p < 0.001), had a higher Human Development Index (HDI, p < 0.001), and were less often diagnosed with amyloidosis (p < 0.001). There was no difference in outcome whether patients were discharged from cardiology or cardiogeriatrics departments. The most frequent precipitating factors underlying AHF decompensation between the first and second hospitalization were arrhythmia and infection.

**Conclusion:**

AHF patients discharged from cardiogeriatrics departments, compared to cardiology departments, showed clinical differences but had the same prognosis regarding AHF rehospitalization at 90 days.

## Introduction

Heart failure is a disease that affects about 2% of the population in western countries [1, 2]. With the aging population, the prevalence of acute heart failure (AHF), measured as the annual incidence of hospitalizations for AHF, rises to more than 10% in patients over 70 years of age [3].

After discharge from the hospital for AHF, readmissions are frequent [4, 5]. Although overall hospitalization rates for heart failure have decreased, unplanned readmissions continue to be a common occurrence, with nearly 30% of patients being readmitted within 90 days of discharge [6, 7]. Among older patients, hospitalization is associated with markedly adverse outcomes, including increased mortality, morbidity, and health care expenditures [8].

Often, heart failure patients are older than 75 years and have other common geriatric conditions including frailty, depression, cognitive impairment, malnutrition, disabilities, and chronic diseases other than a heart condition [9, 10]. The management of these patients depends on geriatrics and cardiology particularities. Cardiologists’ and geriatricians’ awareness and perception of heart failure, comorbidities and functional status can be different and complementary [11]. Indeed, the intervention of cardiologists in the course of care for elderly patients has been shown to improve short-term mortality and readmission outcomes [12, 13]. Calls have been made for a new paradigm in cardiac care for older adults or for closer collaboration between the two specialities [14, 15]. No study has yet established the contribution to HF care of geriatricians with expertise in cardiology.

Some studies have focused on the profile of patients at risk of being rehospitalized [16, 17], and other studies have looked at precipitating factors that trigger acute heart failure [18, 19]. In the context of hospitalization in cardiology and cardiogeriatrics units, the various factors influencing rehospitalization are not clearly established.

We hypothesized that the geriatric conditions of patients hospitalized for AHF could impact the rate and precipitating factors of rehospitalization.

The objectives of this study were (i) to determine the profile and outcome (rehospitalization at 90 days) of patients admitted to Val-de-Marne hospitals for HF, depending on whether they were hospitalized in a cardiology or a specialized cardiogeriatrics department and (ii) to analyze modes and precipitating factors of rehospitalization in the two types of departments.

## Methods

The ICREX-94 research was a prospective, non-interventional, observational, transversal, multicentric registry conducted in seven cardiology units and three cardiogeriatrics units in the Val-de-Marne department (zip code 94) in France.

The present study was conducted in the Val-de-Marne department: 245 sq. km, 1.4 million inhabitants, a mix of residential cities and low-incomes cities (mean HDI 0.58, max 0.78, min 0.35). The Val-de-Marne health system comprises 48 hospitals, with a total capacity of 9500 beds. In 2018, in the 10 hospitals participating in this study, 2393 heart failure admissions were recorded by the “*Caisse Primaire d’Assurance Maladie*” (French health insurance fund), representing 85% of all AHF admissions in Val-de-Marne. In 2016, ten Val-de-Marne cardiology and cardiogeriatrics departments, academic and non-academic, public and private, large and small, interested in HF care, decided to create a network (FINC94) and to collaborate, in order to share their experiences, train healthcare professionals and conduct clinical studies such as this one [20].

There were seven classical cardiology departments, found in both teaching and nonteaching hospitals, public and private, and three cardiogeriatrics departments specialized in heart failure management, with geriatricians who had received specific academic and practical training in cardiovascular medicine. In addition to their geriatrics background, the geriatricians in these units trained for several months in cardiology departments specialized in HF, and have university diplomas in echocardiography and cardiovascular disease of elderly patients. Therefore, they work in close cooperation with the HF team of their departments. There was no specific protocol when patients were hospitalized in cardiology or cardiogeriatrics departments, except to follow 2016 ESC guidelines on HF. Upon admission to cardiogeriatrics departments, an individual and multidisciplinary approach (by geriatricians, physiotherapists, dietitians and social workers) was established, focused on stabilization of comorbidities, return to self-sufficiency and renutrition in addition to specific cardiology follow-up.

There were no specific guidelines to direct patients to a cardiology or cardiogeriatrics department.

Consecutive patients over 18 years of age, hospitalized for acute HF and alive at discharge were eligible for the study. Diagnosis of AHF was based on signs and symptoms of HF— clinical point of view, BNP at admission > 100 pg/ml and heart structure suggesting HF on echocardiograms, as recommended (ESC guidelines). Patients who did not understand the French language were excluded. The study was compliant with Helsinki rules and was approved by the local ethics committee (Commission éthique et déontologie de la Faculté de Médecine Paris-Saclay #20181128163709). All patients gave their informed consent. Informed consent was obtained for all the participants.

### Baseline data collection

The following data was collected at inclusion and if patients were rehospitalized: HF type (i.e., right, left, global), etiology of HF, date of diagnosis of HF, clinical characteristics including geriatric comorbidities like dementia and depression, ECG data (sinus rhythm, atrial fibrillation), and biological data such as BNP, haemoglobinemia, and serum creatinine. In addition, the human development index (HDI), which evaluates the progress of a country or a region in the long term, adapted to the Ile-de-France region, was determined by the town of residency. The HDI takes into account three basic dimensions of human development: a long and healthy life (life expectancy), access to knowledge (education) and a decent standard of living (income) [21]. We recorded echocardiographic characteristics, such as left ventricular ejection fraction (LVEF), and medical treatments with respective doses and whether the patient had a multi-site and/or defibrillator pacemaker. We defined patients as “well-treated” when they had received more than 50% of the target dose of the treatment by ARB and beta blockers.

### Follow-up data collection

Patients were followed over 90 days after discharge from hospital by direct phone calls and correspondence. If the patient did not answer, we called the patient’s family, caregiver, general practitioner or cardiologist. Rehospitalizations within the 90 days were recorded, with medical reports and the same clinical, ECG and biological variables as on first admission. Cause and mode of rehospitalization were analyzed by Clinical Endpoints Committees (CEC) set up to review all medical reports of rehospitalized patients. Each CEC included one cardiologist and one geriatrician trained in endpoint adjudication. All events were reviewed independently by each CEC. Any disagreement between CECs was resolved by a third physician as CEC chairman (EB, KR, LH, CD, TD).

CECs divided hospital admissions in four classes: AHF Planned Rehospitalization, AHF Unplanned Rehospitalization, Non-AHF Planned Rehospitalization, and Non-AHF Unplanned Rehospitalization. For AHF readmissions, the underlying causes were classified by CECs as follows: infection, unstable hypertension, arrhythmia, medical treatment modification, non-adherence, anaemia, myocardial infarction, pulmonary embolism, acute renal failure, very severe chronic heart failure (i.e. “frequent flyer” patients with ≥3 hospitalizations in the year or with NTproBNP > 5000 pg/ml).

### Statistical analysis

Continuous variables are expressed as median [interquartile range (IQR)], and categorical variables are expressed as number or frequency (percentage). Differences in patient clinical characteristics between cardiology and cardiogeriatrics departments were tested by the χ^two^ or fisher test for categorical data and by the Wilcoxon test for continuous data.

Differences in clinical characteristics between patients hospitalized for acute heart failure and non hospitalized patients were obtained with univariate logistic regression and the Wald test.

Finally we produced a Kaplan-Meyer curve of readmission for acute heart failure within 90 days depending on the type of department and did a survival analysis using a univariate cox regression.

A two-sided *p*-value < 0.05 was considered statistically significant. All statistical analyses were performed using R version 4.

## Results

### Characteristics of the study population

Three hundred and eight patients were included between October 2017 and January 2019. Patient characteristics are presented in Table 1. Patients were 75.8 ± 13.5 years old, and 133 (43.3%) were women. Regarding comorbidities, 203 (66.8%) patients had a history of HF, 115 (37.8%) of coronary artery disease, 112 (36.7%) of diabetes, 181 (59.3%) were in atrial fibrillation and 28 (9.2%) had been diagnosed with amyloidosis. Sixty-nine (22.4%) had LVEF > 50% and 186 (60.4%) LVEF < 40%. Mean LVEF was 39 ± 19%. For HF treatment, 259 (85.2%) patients took diuretics, 208 (68.4%) beta-blockers, 183 (59.4%) ACE inhibitors or ARNi or LCZ696, and 60 (20.1%) MRA.

**Table 1:** Baseline characteristics of all patients depending on department (cardiology or cardiogeriatrics)

Department	Overall	Cardiogeriatrics	Cardiology	p
**N (%)**	308	66 (21.4)	242 (78.6)	
**Age**, yrs.	75.8 ± 13.5	82.4 ± 9.2	74.0 ± 13.8	**< 0.001**
**Age classes**, n(%)				**< 0.001**
**< 45** yrs.	9 (2.9)	0 (0)	9 (3.7)	
**45–74** yrs.	125 (40.6)	12 (16.7)	114 (47.1)	
**75–85** yrs.	98 (31.8)	26 (39.4)	72 (29.7)	
**> 85** yrs.	76 (24.6)	28 (42.4)	48 (19.8)	
**Female Gender**, n(%)	133 (43.3)	32 (49.2)	101 (41.7)	
**Live alone**, n(%)	54 (17.5)	24 (36.4)	30 (12.4)	**< 0.001**
**Married**, n(%)	70 (22.7)	14 (21.2)	56 (23.1)	0.869
**Live with family**, n(%)	31 (10.1)	6 (9.1)	25 (10.3)	1
**Live in institution**, n(%)	3 (1)	3 (4.5)	0	**0.009**
**IDH**	0.6 ± 0.1	0.6 ± 0.1	0.54 ± 0.1	**< 0.001**
**Coronary heart disease**, n(%)	115 (37.8)	23 (35.4)	92 (38.5)	0.668
**Amyloidosis**, n(%)	28 (9.2)	1 (1.5)	27 (11.2)	**0.014**
**Hypertension**, n(%)	207 (67.9)	44 (67.7)	163 (67.9)	1
**Diabetes**, n(%)	112 (36.7)	17 (26.2)	95 (39.6)	0.059
**Smoker**, n(%)	38 (12.5)	8 (12.3)	30 (12.5)	1
**Chronic alcohol intake**, n(%)	23 (7.5)	5 (7.7)	18 (7.5)	1
**Chronic kidney disease**, n(%)	141 (46.2)	27 (41.5)	114 (47.5)	0.404
**COPD**, n(%)	65 (21.3)	14 (21.5)	51 (21.2)	1
**History of heart failure**, n(%)	203 (66.8)	43 (66.1)	160 (66.9)	1
**Stroke**, n(%)	47 (15.4)	13 (20)	34 (14.2)	0.25
**Atrial fibrillation**, n(%)	181 (59.3)	38 (58.5)	143 (59.6)	0.888
**Dementia**, n(%)	24 (7.9)	20 (13.6)	11 (4.68)	**< 0.001**
**Depression**, n(%)	15 (4.9)	9 (13.6)	6 (2.5)	**0.001**
**BMI**, kg/m2	27.1 ± 7.2	26.2 ± 6.6	27.2 ± 7.3	0.03
**SBP**, mmHg	122 ± 20	122 ± 18	123 ± 22	0.855
**LVEF classes**, n(%)				0.584
**> 50%**	69 (22.4)	13 (19.7)	56 (23.1)	
**40–50%**	53 (17.2)	14 (21.2)	39 (16.1)	
**< 40%**	186 (60.4)	39 (59.19)	147 (60.7)	
**BNP at discharge, pg/ml**	554 ± 745	618 ± 823	300 ± 112	**0.001**
**Length of stay**, days	12.7 ± 10.1	19.3 ± 15.4	11.2 ± 7.3	**< 0.001**
**Creatinemia at discharge**, days	134 ± 63	118 ± 38	137 ± 67	0.18
**Prescription at discharge**, n(%)	292 (95.74)	63 (95.45)	229 (95.82)	0.254
**ACEi**, n(%)	133 (43.9)	34 (53.1)	99 (41.4)	0.118
**ACEisup50**, n(%)	53 (17.5)	12 (18.7)	41 (17.1)	0.853
**ARNi**, n(%)	25 (8.2)	5 (7.8)	20 (8.3)	1
**ARNisup50**, n(%)	13 (4.3)	5 (7.8)	8 (3.3)	0.157
**Diuretic**, n(%)	259 (85.2)	51 (79.7)	208 (86.7)	0.169
**Dose Diuretic**, n(%)	118 ± 190	71 ± 117	131 ± 204	**0.002**
**Beta blocker**, n(%)	208 (68.4)	46 (71.9)	162 (67.5)	0.548
**Beta blockersup50**, n(%)	83 (27.3)	14 (21.9)	69 (28.7)	0.344
**MRA**, n(%)	61 (20.1)	7 (10.9)	54 (22.5)	0.052
**MRAsup50**, n(%)	45 (14.8)	6 (9.4)	39 (16.2)	0.234
**Ivabradine**, n(%)	7 (2.3)	2 (3.1)	5 (2.1)	0.641
**LCZ696**, n(%)	25 (8.2)	5 (7.8)	20 (8.3)	1
**Pacemaker**, n(%)	41 (13.4)	16 (24.2)	25 (10.4)	**0.007**
**ICD**, n(%)	43 (14.1)	2 (3)	41 (17.1)	**0.002**
**Well Treated**, n(%)	24 (7.9)	36 (56.2)	118 (49.2)	0.328

Note. The data are quoted as the number (%), or mean ± sd, "well treated" was defined when patients were treated with BB+ACEi or ARNi

*Abbreviations*: *ACEi* Angiotensin-Converting Enzyme inhibitors, *ARNi* Angiotensin Receptor Neprilysin inhibitor, *BMI* Body Mass Index, *COPD* Chronic Obstructive Pulmonary Disease, *HDI* Human Development Index, *HF* Heart Failure, *HR* Heart Rate, *ICD* Implantable Cardioverter Defibrillator, *MRA* Mineralocorticoid Receptor Antagonists, *SBP* Systolic Blood Pressure, *LVEF* Left Ventricular Ejection Fraction

### Characteristics of patients hospitalized depending on the department

The clinical profiles of AHF hospitalized patients depending on the department (cardiogeriatrics or cardiology) are shown in Table 1. In cardiogeriatrics units, they were older (*p* < 0.001) and less often diagnosed with amyloidosis (p < 0.001), and had higher BNP levels at discharge (p < 0.001) and longer stays (p < 0.001). They more frequently lived alone (p < 0.001) or in institutions (p < 0.001), with a major neurocognitive disorder (p < 0.001) or depression (p < 0.001). However, their HDI was higher (p < 0.001). LVEF was higher in cardiogeriatrics departments (43 ± 14% vs 36 ± 19%). Regarding treatment, patients from cardiogeriatrics departments took lower doses of diuretics (*p* = 0.002), and more frequently had an implanted pacemaker (*p* = 0.007) and less frequently an Implantable Cardioverter-Defibrillator (ICD) (p = 0.002) (Table 1). There was no difference between cardiogeriatrics and cardiology units in the prescription of angiotensin-converting enzyme inhibitors (ACEI), angiotensin II receptor blockers (ARBs) or beta-blockers (BB-).

### Patient outcomes at 90 days

**Fig. 1:**
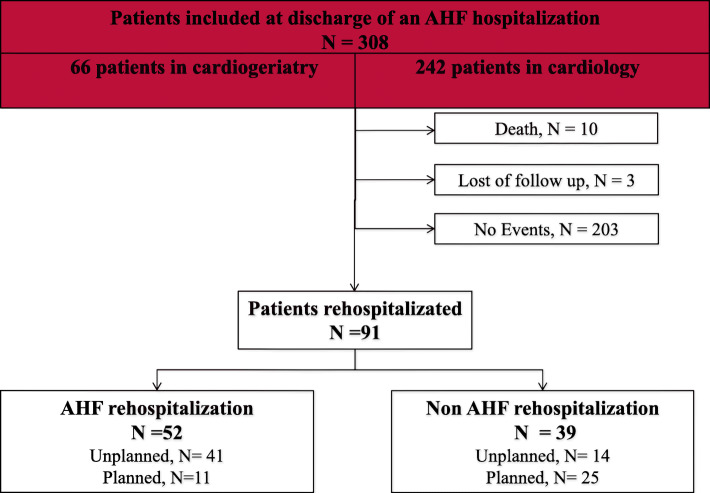
Flow chart

**Fig. 2:**
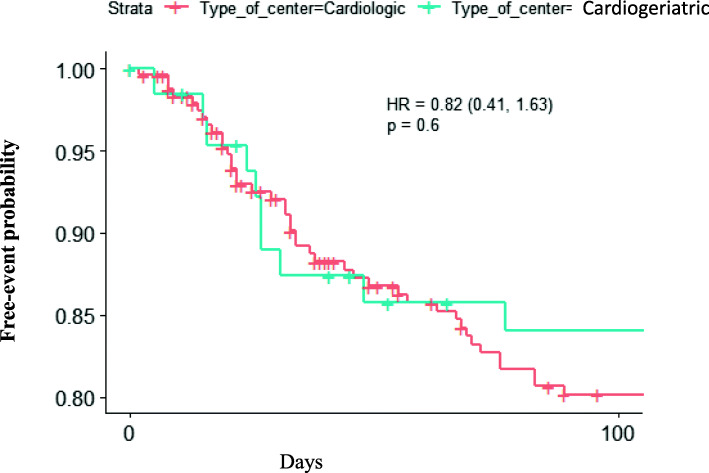
Patients’ outcome at 90 days according to the type of department, cardiologic or cardiogeriatric: AHF planned or unplanned

At 90 days, of the 308 patients discharged from hospital, 52 (17%) were readmitted for AHF and 39 (12.6%) for non-cardiovascular causes (Fig. 1). Of the 52 readmitted for AHF, 41 (13.3%) were unplanned and 11 (3.6%) planned readmissions. Of the 39 readmitted for other reasons, 25 (8.1%) were planned, and 14 (4.5%) unplanned non-AHF rehospitalization. Regarding the department of discharge, cardiogeriatrics or cardiology, there was no statistical difference in the primary endpoint “90 days rehospitalization for AHF” (Fig. 2). Regarding death at 90 days, there were 22 deaths in patients discharged from cardiologic department and 2 deaths in patients discharged from cardiogeriatric department, with no statistical difference (*p* = 0. 146).

**Fig. 3:**
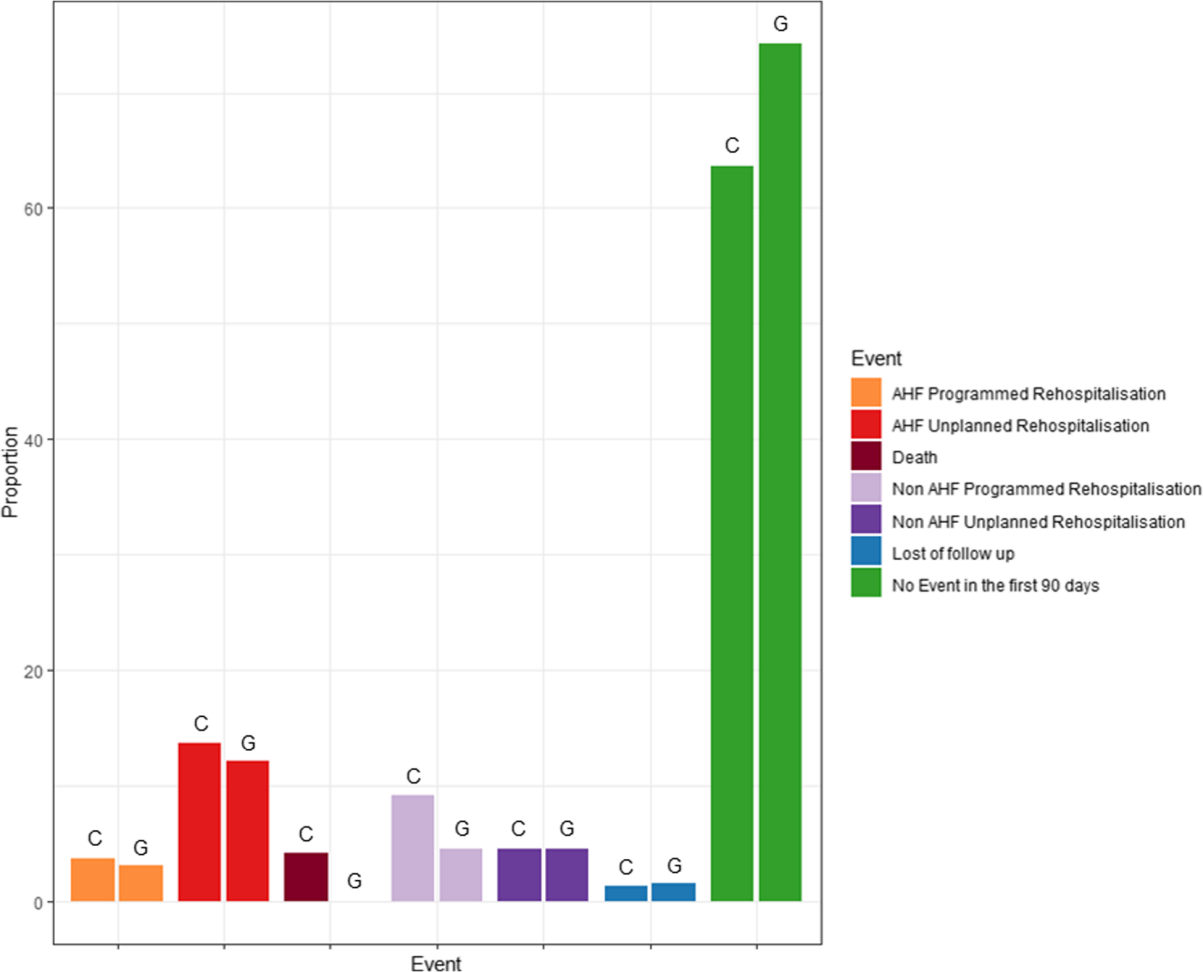
Patients’ outcome at 90 days according to the type of department cardiologic (C) or cardiogeriatric (G)

Compared with cardiology departments, when patients had been initially hospitalized in cardiogeriatrics departments, there was no statistical difference regarding the type of rehospitalization (AHF unplanned, AHF planned, non-AHF planned, non-AHF unplanned) (Fig. 3 and Table 2).

**Table 2:** Events in the population following discharge, depending on department

Type of Center	Overall	Cardio-geriatrics	Cardiology	p
**N**	308	66	242	
**Reasons and mode of rehospitalization, n(%)**				0.53
**AHF Unplanned**, n(%)	41 (13.3)	8 (12.1)	33 (13.6)	
**AHF Planned**, n(%)	11 (3.6)	2 (3.0)	9 (3.7)	
**Non-AHF Planned**, n(%)	25 (8.1)	3 (4.5)	22 (9.1)	
**Non-AHF Unplanned**, n(%)	14 (4.5)	3 (4.5)	11 (4.5)	
**Number of events in the first 90 days**, n(%)	91 (29.5)	16 (24.2)	75 (31)	0.11

Note. The data are quoted as the number (%)

*Abbreviations*: *AHF* Acute Heart Failure

### Risk of AHF rehospitalization according to causes

**Fig. 4:**
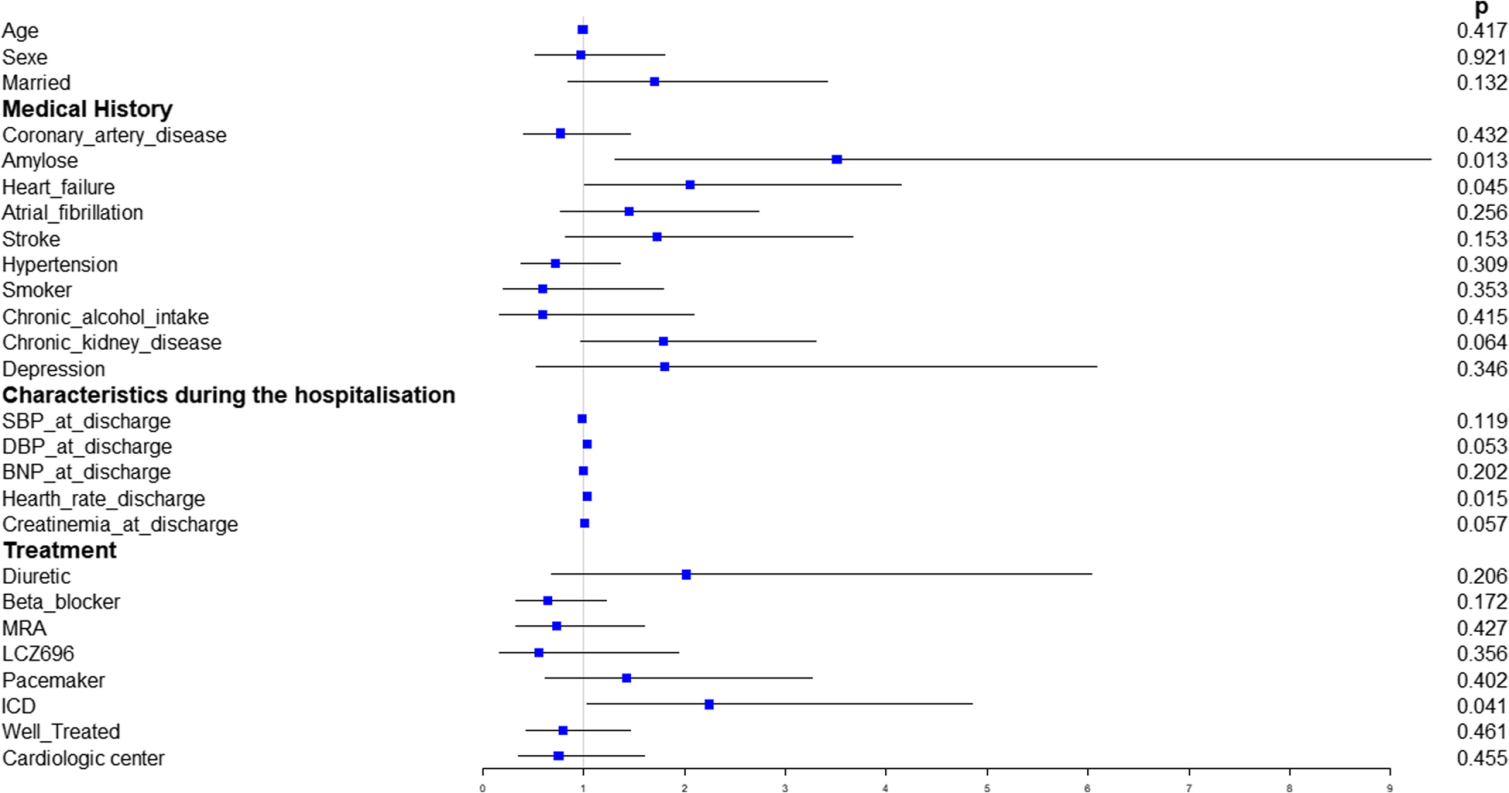
Forest plot of different parameters influencing AHF hospitalization in the overall population

In univariate analysis, after a first AHF rehospitalization, the profiles of patients at risk of being rehospitalized for AHF in our study were analyzed. A past history of HF, amyloidosis, high Heart Rate (HR) at discharge and intracardiac defibrillator usage was associated with a higher risk of AHF rehospitalization at 90 days (Fig. 4).

### Causes of hospitalization in HF patients by department: first and second hospitalization in the 90-day follow-up

**Fig. 5:**
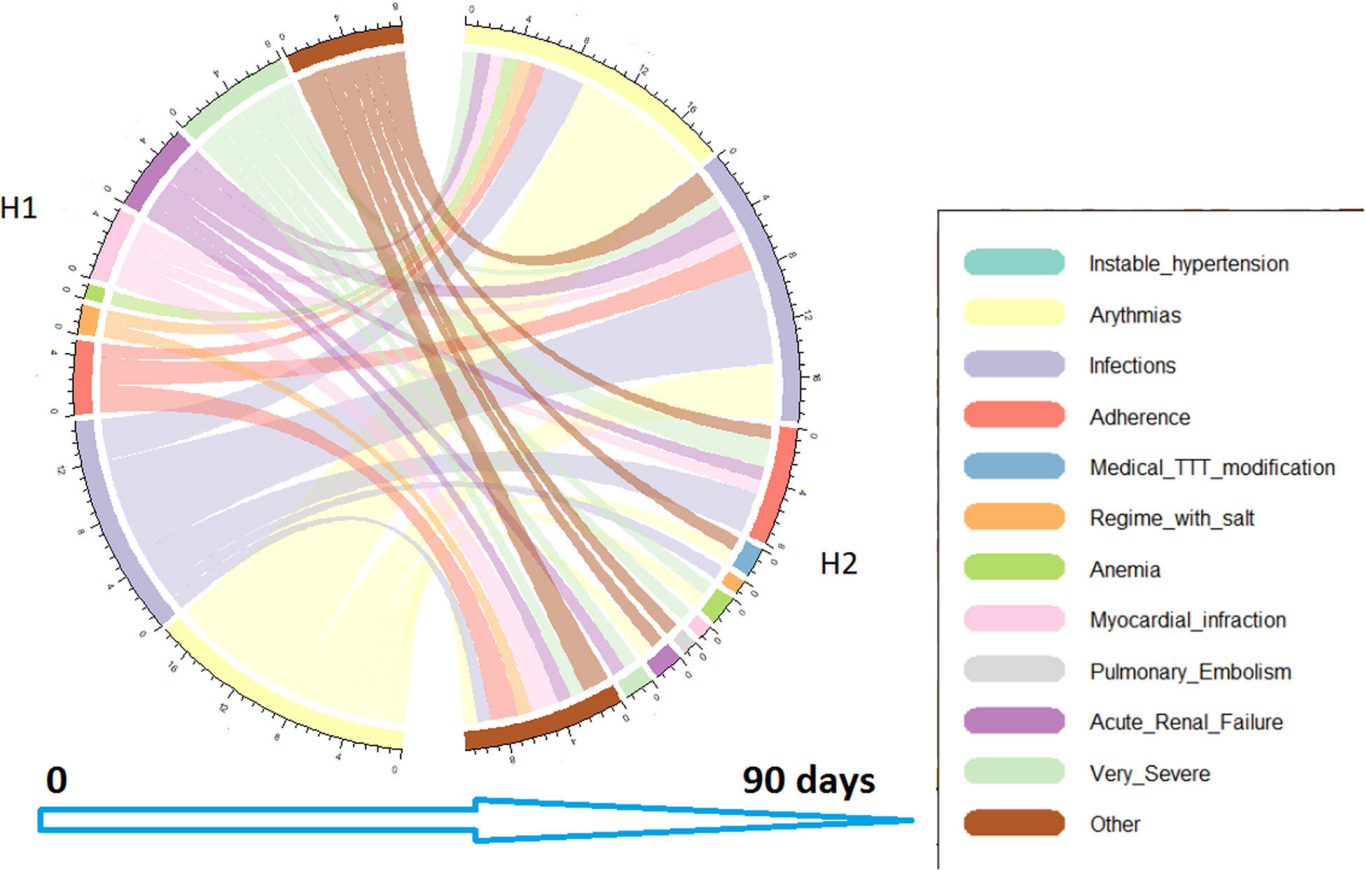
Relationship in main Causes of hospitalisation in AHF Patients at 90 days between hospitalisation (H1) and rehospitalisation (H2)

The most frequent precipitating factors for AHF decompensation between the first and second hospitalizations (H1 and H2) were arrhythmia (42.3%), infection (30.8%) and very severe symptoms (17.3%) (Table 3 and Fig. 5). When analyzing differences between cardiogeriatrics or cardiology admissions, we found no difference in the causes for hospitalization, which were predominantly arrhythmia and infection.

**Table 3:** Causes of rehospitalization of HF patients depending on department: first rehospitalization (H1) and second rehospitalization (H2) during 90-day follow-up

Type of Center	Overall	Cardio-geriatrics	Cardiology	p
**H1: First hospitalization**				
**Arrythmias**, n(%)	88 (28.6)	15 (22.7)	73 (30.2)	0.283
**Infection,** n(%)	76 (24.7)	17 (25.8)	59 (24.4)	0.872
**Poor adherence to TT**, n(%)	17 (5.5)	0 (0)	17 (7.0)	**0.029**
**Medical TT modification**, n(%)	2 (0.6)	1 (1.5)	1 (0.4)	0.383
**Poor diet observance**, n(%)	26 (8.4)	3 (4.5)	23 (9.5)	0.316
**Anemia**, n(%)	18 (5.8)	7 (10.6)	11 (4.5)	0.076
**Myocardial infarction**, n(%)	22 (7.1)	6 (9.1)	16 (6.6)	0.589
**Pulmory Embolism**, n(%)	2 (0.6)	0 (0)	2 (0.8)	1
**Acute Renal Failure**, n(%)	23 (7.5)	2 (3.0)	21 (8.7)	0.184
**Very Severe HF**, n(%)	8 (2.6)	1 (1.2)	7 (2.9)	1
**Unstable hypertension**, n(%)	6 (1.9)	2 (3.0)	4 (1.6)	0.612
**Other**, n(%)	60 (19.5)	15 (22.7)	45 (18.6)	0.484
**H2: Second hospitalization**				
**Arrythmias**, n(%)	28 (21.9)	4 (23.5)	24 (21.6)	1
**Infections**, n(%)	28 (21.9)	2 (11.7)	26 (23.4)	0.36
**Poor adherence to treatment**, n(%)	8 (6.2)	0 (0)	8 (7.2)	0.596
**Medical TT modification,** n(%)	2 (1.6)	0 (0)	2 (1.8)	1
**Poor regimen adherence**, n(%)	6 (4.7)	1 (5.9)	5 (4.5)	0.583
**Anemia**, n(%)	5 (3.9)	2 (11.8)	3 (2.7)	0.131
**Myocardial infarction**, n(%)	3 (2.3)	0 (0)	3 (2.7)	1
**Pulmory Embolism**, n(%)	1 (0.8)	0 (0)	1 (0.9)	1
**Acute Renal Failure**, n(%)	7 (5.5)	1 (5.9)	6 (5.4)	1
**Very Severe HF**, n(%)	2 (0.6)	0 (0)	2 (0.8)	1
**Other** n(%)	38 (29.7)	8 (47.1)	30 (27)	0.151

Note. The data are quoted as the number (%)

*Abbreviations*: *TT* Treatment, *HF* Heart Failure

## Discussion

In a multicentric study in the Val-de-Marne area south-east of Paris, we prospectively conducted a comprehensive assessment of AHF patient profiles and the modes and causes of rehospitalization within 90 days, depending on whether patients were hospitalized in cardiogeriatrics or cardiology departments.

To our knowledge, this is the first study comparing patients hospitalized in cardiology vs. cardiogeriatrics with specific geriatrician training.

Our study had two main findings:

- While AHF patients in cardiogeriatrics were older, less independent, less often diagnosed with amyloidosis, more often living alone, more often with major neurocognitive disorder or depression, but with higher HDIs, there was no statistical difference in the primary endpoint “hospitalization for AHF” depending on the specialty department of discharge.
- The most frequent precipitating factors underlying AHF decompensation between the first and second hospitalization were arrhythmia, infection or very severe symptoms, and it made no difference whether patients were discharged from cardiogeriatrics or cardiology units.

### Readmission for HF

The characteristics of our population are similar to those in previous reports on the general population in terms of age, gender, risk factors, coronary artery disease, diabetes mellitus, atrial fibrillation, prescription of diuretics, BB-, ACE-I/ARB/ARNi and MRA [19, 22].

In our study, the readmission rate at 90 days was 29.6%, comparable to rates previously reported for the same timeframe [6, 7]. We chose to present data at 90 days because the restricted 30-day window has been questioned. Readmissions for HF are a real problem. Strategies intended to reduce rates of premature admission have been developed. Good stabilization of HF can reduce the occurence of readmission [6, 23]. In addition, data comparing the relative utility of a 30-day window versus other post-discharge timeframes showed limited differences in assessing overall hospital performance [24].

While previous studies demonstrated worse outcomes in elderly patients [24], in the present study at 90 days there was no difference in the rate of readmission for AHF nor of death (Fig. 3). In our study, cardiogeriatrics patients were older, had higher rates of depression and neurocognitive disorders, and lived more frequently alone or in an institution. They had higher BNP levels. Post discharge, they received a lower dosage of diuretics and were more frequently implanted with a pacemaker and less frequently with an ICD. The absence of difference in the rehospitalization rate of patients from cardiogeriatrics departments vs. cardiology departments may be partly due to the similarity in maintenance therapy (BB-, ACE, i/ARNi), and cardiogeriatrics patients’ higher HDI, which may counterbalance the effects of age, dementia or depression on the rate of readmission [25].

### Management of elderly HF patients

A recent study concluded that a “cardiogeriatrics model” of managing HF did not improve the prognosis of HF patients at 30 days [26]. There is a room for innovative care for elderly HF patients [15, 16]. Though cardiogeriatrics patients were older and more socially isolated and dependent, with more mood disorders and major neurocognitive disorders, there was no significant difference in rates of readmission for AHF at 90 days compared to cardiology patients. This similarity in prognosis may be linked to a similar efficacy in therapeutic management, a comprehensive and specific multidisciplinary approach and to a longer stay in cardiogeriatrics departments allowing for better stabilization of comorbidities that may lead to rehospitalization [27]. It is also possible that the recruitment and care of our elderly patients through cardiogeriatrics departments may differ from usual geriatrics departments. Indeed, the cardiogeriatrics departments are characterized by geriatricians with specific competences in cardiology, with easy access to echocardiography and BNP measurement, and in close contact with cardiologists in the cardiology departments of our area.

Comprehensive patient management seems essential to reduce readmissions and thus improve the quality of life of these patients. Achieving this outcome will require training cardiologists to manage multiple morbidities and frailty, and improving the skills of geriatricians in HF management [15, 16]. The use of a frailty score accessible to cardiologists will facilitate the collaboration between cardiologists and geriatricians within the heart team serving the patient [27].

#### Risk of acute heart failure hospitalization according to profile and causes

In our population, the factors associated with AHF readmissions were: previous AHF history, higher HR at discharge, cardiac amyloidosis, and intracardiac defibrillator use. These factors have previously been shown to influence HF readmissions [17]. Precipitating factors and their contribution to hospitalization of patients with HF have been previously described [7, 18, 19]. In the present study, according to those reports, factors that influenced the most readmissions were infection, cardiac arrhythmia and severity of heart failure. Interestingly, when precipitating factors were analyzed for the second readmissions, factors remained similar for some patients, but differed for others, showing the complexity and heterogeneity of the heart failure process in different patients. Moreover, the same frequent causes were found in both cardiology and cardiogeriatrics departments.

### Limitations

The present study has several limitations.

The study was performed in the Val-de-Marne department of France, and therefore may have limited implications for other territories with different environments or healthcare systems. The numbers of patients recruited in cardiology and cardiogeriatrics departments were not evenly balanced, thus limiting the strength of our results; however, the data was recorded prospectively over the same period of time. The percentage of patients with preserved ejection fraction appears lower than usually observed in elderly patient studies [26, 28]. Due to the mode of recruitment, our study includes relatively few patients and may lack power. In a future work, the patient cohort could be larger, better distributed between the admissions departments. Results could include mortality, and more diverse geriatric outcomes, such as functional outcomes, necessity of (nursing) home care after first admission or patient satisfaction data.

Some elements that could explain rehospitalizations were not noted (multidomain assessment of frailty) and could be the subject of future work.

## Conclusion

While clinically different, AHF patients discharged from cardiogeriatrics compared to cardiology departments, had similar prognosis regarding rehospitalization for AHF at 90 days. Among other possibilities, care provided in cardiogeriatrics departments by geriatricians with cardiology/HF expertise may have played a role, suggesting the effectiveness of innovative management of elderly HF patients. The main precipitating factors underlying AHF decompensation for the first rehospitalization were arrhythmia, infection, and very severe symptoms, in both cardiology and cardiogeriatrics departments, and remained proportionally similar during the second hospitalization. Further studies are needed to confirm these conclusions.

## Data Availability

Data and materials are available: Raw data are available on the following link: https://drive.google.com/file/d/1bHDRR_Z0PDXPoulMZG08E-9AtFKM7ryR/view?usp=sharing

## Abbreviations

ACEi: Angiotensin-Converting-Enzyme inhibitors
ARNi: Angiotensin Receptor Neprilysin inhibitor
BMI: Body Mass Index
COPD: Chronic Obstructive Pulmonary Disease
HDI: Human Development Index
HF: Heart Failure
HR: Heart Rate
ICD: Implantable Cardioverter-Defibrillator
MRA: Mineralocorticoid Receptor Antagonists
SBP: Systolic Blood Pressure
LVEF: Left Ventricular Ejection Fraction
